# Self-Standing, Ultrasonic Spray-Deposited Membranes for Fuel Cells

**DOI:** 10.3390/membranes13050522

**Published:** 2023-05-17

**Authors:** Ali Karaca, Irina Galkina, Yoo Jung Sohn, Klaus Wippermann, Fabian Scheepers, Andreas Glüsen, Meital Shviro, Martin Müller, Marcelo Carmo, Detlef Stolten

**Affiliations:** 1 Institute of Energy and Climate Research (IEK-14): Electrochemical Process Engineering, Forschungszentrum Jülich, Wilhelm-Johnen-Straße, 52428 Jülich, Germanymeital.shviro@nrel.gov (M.S.);; 2 Institute of Energy and Climate Research (IEK-1): Materials Synthesis and Processing, Forschungszentrum Jülich, Wilhelm-Johnen-Straße, 52428 Jülich, Germany; 3 Institute of Energy and Climate Research (IEK-3): Techno-Economic Systems Analysis, Forschungszentrum Jülich, Wilhelm-Johnen-Straße, 52428 Jülich, Germany; 4 Chair for Fuel Cells, RWTH Aachen University, 52072 Aachen, Germany

**Keywords:** membrane, spray deposition, fuel cells, DMFC, permeability, membrane structure, ultrasonic, drying temperature, high boiling solvent, water electrolysis

## Abstract

The polymer electrolyte membrane and its contact with electrodes has a significant effect on the performance of fuel and electrolysis cells but the choice of commercially available membranes is limited. In this study, membranes for direct methanol fuel cells (DMFCs) were made by ultrasonic spray deposition from commercial Nafion solution; the effect of the drying temperature and presence of high boiling solvents on the membrane properties was then analyzed. When choosing suitable conditions, membranes with similar conductivity, water uptake, and higher crystallinity than comparable commercial membranes can be obtained. These show similar or superior performance in DMFC operation compared to commercial Nafion 115. Furthermore, they exhibit low permeability for hydrogen, which makes them attractive for electrolysis or hydrogen fuel cells. The findings from our work will allow for the adjustment of membrane properties to the specific requirements of fuel cells or water electrolysis, as well as the inclusion of additional functional components for composite membranes.

## 1. Introduction

Fuel cells and water electrolysis can make a major contribution to decarbonizing the energy system, one of the major challenges of today’s society. The success of fuel cells partly depends on individual components such as catalysts, membranes, or gas diffusion layers. Each of them is crucial for a cell’s performance and efficiency. However, it is not sufficient to consider each component in itself, but rather to see them as parts of a unit that must be suited to each other. Catalyst-coated membranes (CCMs) or membrane–electrode assemblies (MEAs) can be such units. A main component of a CCM is the polymer–electrolyte membrane (PEM) that undertakes several roles within an electrochemical cell, working as an electronic separator but enabling protonic conductivity [1]. They are responsible for mechanical stability [2] and serve as the main barrier against fuel crossover [3]. Based on this, the main aim of CCM manufacturing should be to produce CCMs with high catalyst utilization and high protonic conductivity while ensuring low fuel crossover, resulting in high levels of performance and efficiency. Currently, there are several ways to manufacture CCMs. One method is to coat a PTFE-based substrate with an electrode material and transfer it to a membrane by means of hot-pressing [4]. This process is employed for either lab-scale production (e.g., doctor-blade coating and decal transfer) [5] or large-scale manufacturing (e.g., slot-die coating and roll-to-roll) [6]. The main challenge is to guarantee a complete transfer of electrode material [7] while avoiding gaps between the membrane and electrode. Another approach would be to coat the gas diffusion layers (GDLs) with electrode material (GDE) and subsequently hot-press it onto the membranes (MEA) [8,9,10]. As electrode ink can also penetrate into the GDL, catalyst utilization can be lower than with CCMs [11]. Finally, there are several ways to directly coat a membrane by either spray deposition or doctor-blade coating onto a heated and fixed membrane [12,13,14], or onto a pre-swollen membrane [15,16]. Both approaches can be critical because of the swelling behavior of Nafion [15]. If coated by means of spray deposition, heating and fixing by vacuum must be ideal in order to avoid local swelling or bubbles in the membrane. If pre-swollen, it must be ensured that the membrane maintains the degree of swelling during the entire coating procedure so as to avoid inhomogeneities [15].

Even though all of these processes work, they rely on commercial membranes such as Nafion. In our view, this represents a major restriction when approaching CCM fabrication as a unit rather than an accumulation of several components. A solution to this problem would be the integration of membrane fabrication as part of CCM manufacturing. Several steps have been taken in favor of this approach in the literature. Klingele et al., for instance, have developed the direct membrane deposition (DMD) technique whereby Nafion solutions are spray deposited onto gas diffusion electrodes (GDE) [17,18]. Bayer et al. employed the same technique to produce electrode-supported MEAs with membranes as thin as 10 µm [19]. All of these approaches are promising, but in the same way that decal transfer processes rely on membrane foils, the spray deposition of ionomer solutions onto electrodes relies on GDL materials and their properties. To combine any electrode with any given GDL or PTL material, the membrane should be fabricated along the CCM production process as a self-standing membrane, independent of the electrode support. This route was presented by Stähler et al. where the anode, membrane, and cathode were consecutively slot-die-coated [20]. The combination of an approach chosen by Stähler et al. with ultrasonic spray deposition for membrane fabrication offers the maximum freedom for CCM production because, with spray deposition, there are only a few limitations to modify the thickness of any layer or geometry of the required CCM without any hardware modification. Some properties of the membranes can be influenced by thermal treatment [21,22,23] or the addition of high-boiling point solvents [22,24], as these can influence ionomer solution structure [25,26]. Thus, in this work, we aim to produce Nafion-based polymer electrolyte membranes with several modifications: Untreated and thermally treated after production and produced with additional high-boiling point solvents. The hypothesis of this paper is that by controlling the manufacturing and annealing parameters, it is possible to control the properties of the membranes. All membranes will be independent of electrode or GDL support. This method is highly versatile, as it is only based on raw products such as catalyst powders or ionomer solutions. We aim to lay the foundation for a quick transfer to other electrochemical systems (e.g., PEMFCs, PEMWEs, or ammonia cell reactors) and create the opportunity for novel cell designs regarding the membrane (e.g., interlayers, composite membranes, or sandwich structures).

## 2. Materials and Methods

### 2.1. Chemicals and Materials

LQ-1115 Nafion^®^ ionomer solution (EW 1100, 15% wt. Nafion, 45% wt. Propanol, 40% wt. water) was purchased from Ion Power Nafion Store™ and used as received. PtNi nanoparticles (4–5 nm; 25% mol Ni, 75% mol Pt) on Vulcan XC72 (33% wt. PtNi on 67% wt. Vulcan XC72) were also used as received from the Fraunhofer Institute for Applied Polymer Research (Center for Applied Nanotechnology CAN) [27]. All electrode ink formulations were based on deionized water (18 MΩcm) from a Millipore water purification plant. n-Propanol (99.7%) was purchased and used as received from Merck. Glass fiber-reinforced PTFE films (Reichelt Chemietechnik GmbH & Co. (Heidelberg, Germany)) were used as decal transfer sheets. Freudenberg paper (H2315 CX167) was used as the cathode GDL.

### 2.2. Fabrication Techniques

**Nafion solution.** For this study, two types of Nafion solutions were prepared. For Nafion solution type I, a stock Nafion solution (15% wt.) was diluted with a mixture of deionized water and 2-propanol to a Nafion content of 2% wt. with a solvent mass ratio of 47% wt. water and 53% wt. 2-propanol. Nafion solution type II was prepared by diluting the same stock solution of Nafion down to 2% wt. as well, but additional solvents, such as DMSO, DMF, DMAc, or EG, were also added. The final mixture had a composition of 2% wt. Nafion, 2% wt. of additional solvent and 96% wt. of water/2-propanol-mixture.

**Electrode ink**. The required amount of catalyst powder was weighed in a mixing vessel. Furthermore, water, 1-propanol, and the ionomer solution were added to the catalyst powder in that order to avoid a spontaneous ignition of the catalyst powder. Everything was mixed with an ULTRA-TURRAX^®^ Tube Drive P control workstation (4000 rpm; 1 min) and subsequently treated in an ice-cooled ultrasonic bath (20 min) to produce a smooth catalyst dispersion.

**Spray Deposition Process**. All ultrasonic spray depositions were conducted with a Sonotek Exacta Coat device. First, the membrane solution was filled into a syringe pump system. A substrate was placed under the spray head (nozzle) and held down by vacuum on a porous metal plate that was heated to 110 °C to enable quick drying of the spray-deposited membrane solution. The membrane solution was then ultrasonically sprayed onto the substrate (1.5 mL/min, 120 kHz). The membrane thickness was controlled to be the same as Nafion 115 through the number of layers of spray-deposited Nafion solution. In the case of Nafion solution type I, the membrane was either used directly or thermally treated with different temperatures (135, 165, or 195 °C) for 1 h and then removed from the oven to room temperature. In the case of Nafion solution type II, the membrane was produced at 130 °C, even though the additional solvents have higher boiling points. The Nafion/Additional Solvent-Ratio was 1:1 (gravimetrically). Following the deposition process, the membrane was kept in a vacuum furnace at 130 °C and at the vapor pressure of the specific additional solvent, so as to fully dry it. After the membrane fabrication and thermal treatment, the catalyst ink, which had been filled into a second syringe pump system, was ultrasonically spray deposited onto the substrate with the second nozzle (0.3 mL min^−1^; 25 kHz). The catalyst loading was controlled through the number of layers applied. The cathodes were then deposited directly onto the membrane.

**MEA Fabrication**. Following the fabrication of the membrane and deposition of the cathode onto the membrane, anode GDEs were hot-pressed at 130 °C for 3 min at 0.5 kN/cm^2^ to produce an MEA. For this, PtRu (HiSpec12100)-based electrode ink was applied to a Freudenberg GDL (H2315) by slot-die coating as described in a previous paper [28]. The resulting GDE (3.0 mg cm^−2^ PtRu) was then used for the hot-pressing procedure (130 °C, 0.5 kN cm^−2^, 3 min^)^. The cathode GDL was manually placed onto the cathode while assembling the single cell test hardware. The cell geometry was 42 × 42 mm, which corresponds to a geometric surface area of 17.64 cm^2^.

### 2.3. Characterization Methods

**Solvation Test.** All membrane types were weighed, kept in a methanol solution (0.75 mol L^−1^, 1 h, 70 °C), dried (1 h, 60 °C), and weighed again to check if the membranes had dissolved.

**Swelling Test**. All membrane types were also soaked in water (24 h; RT), and weighed and measured before and after to determine the degree of swelling.

**Optical Microscopy (OM)**. For all microscopic investigations, a Zeiss Axio Imager ML optical microscope with 10×/0.25 HD, 5×/0.13 HD and 2.5×/0.06 HD EC Epiplan-NEOPLOAR objectives (25× to 100× magnification) and an AxioCam MR Rw3 camera was used.

**Scanning Electron Microscopy (SEM)**. All samples were analyzed with a Zeiss, Gemini Ultra Plus Scanning Electron Microscope, and an Oxford Instruments (High Wycombe, UK), Ultim Max 100 EDX-Detector.

**Electrochemical Tests.** All electrochemical tests were carried out in homemade test cells with an active surface area of 42 × 42 mm^2^. Checkerboard flow fields machined from carbon plates in the dimension of 1-1-1 mm (width of lands and channels, depth of channels) were used to ensure minimal pressure drop.

**Linear Sweep Voltammetry (LSV).** Experiments were carried out at 70 °C with an electrochemical workstation (IM6) from Zahner-Elektrik GmbH & CoKG to determine hydrogen permeability. The anode was purged with hydrogen (80% RH; 200 mNL min^−1^) and the cathode with nitrogen (80% RH; 50 mNL min^−1^) until the cell potential reached equilibrium at 0.1 V. A linear sweep was then applied to the cathode (0–0.6 V; ν = 5 mV s^−1^).

**Electrochemical Impedance Spectroscopy (EIS)**. EIS measurements were conducted at 70 °C using the above-mentioned IM6. Impedances were measured with a half-cell measurement set-up. The anode was provided with methanol (0.75 mol L^−1^) and the cathode was purged with hydrogen (80% RH) (DHE). All measurements were carried out in potentiostatic mode (0.3 V) with a frequency range from 1 Hz to 50 kHz and a sinusoidal amplitude of 10 mV. All EIS simulations for data-fitting were performed with the Thales EIS simulation program from Zahner-Elektrik GmbH & CoKG. The measurements were fed into the simulation program by using a Hilbert transformation to dismiss possible measurement artifacts. The data were then fitted by using an equivalent electrical circuit (EEC) to determine the ohmic resistances of the fabricated membranes.

**X-ray Diffraction (XRD)**. XRD measurements were conducted with an X-ray diffractometer D8 DISCOVER from Bruker. For the measurements, the diffractometer was operated with a Cu-Kα monochromatic beam with a wavelength of 0.154 nm, 40 kV, and 40 mA. The measurement range was 4–80° in 2θ with 0.01° steps and a measurement pace of 1° s^−1^.

**Performance Tests**. All MEAs were tested with an electronic load from Höcherl und Hackl (ZS506-4 with the zero-volt option). The anode was fed with methanol solution (0.75 mol L^−1^; 3.88 mL min^−1^) and the cathode with dry air (650 mL min^−1^) at sea level pressure. While methanol solution was preheated to 70 °C, the air was neither preheated nor humidified. All single cell tests were carried out at 70 °C using external heating. Each current step was held for 3 minutes, and the average voltage of the last 90 s was reported.

## 3. Results and Discussion

Eight different types of spray-deposited membranes were fabricated and divided into two groups. Membranes were produced with a water–alcohol solution and then thermally treated (Group 1) and membranes produced with additional high-boiling point solvents (Group 2). Further details can be found in the experimental section. In the following, the samples are labeled as M.(T) or M.(S)—where T means the temperature applied for annealing and S the additional solvent used to fabricate the membrane. All attempts to produce EG-based membranes resulted in samples with defects, holes, and rips during the fabrication process. Therefore, this is not discussed in the following.

**Figure 1:**
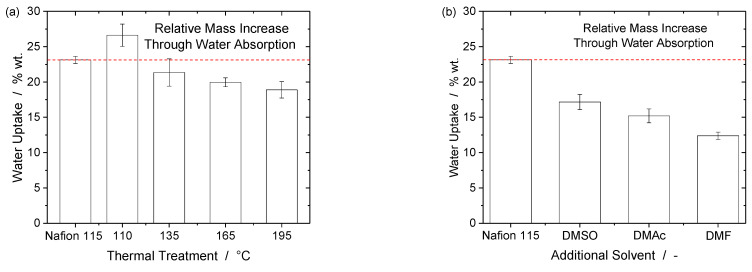
Water uptake of commercial Nafion 115 and all in-house-fabricated membranes: (**a**) M(110)-M(195); and (**b**) M(DMSO), M(DMAc), and M(DMF). The higher the annealing temperature, the smaller the water uptake values. The lower the boiling points of additional solvents, the lower the relative water uptake. The dotted line shows the level of Nafion 115 to facilitate comparison.

Before any kind of analysis can be conducted, it must be ensured that the fabricated membranes do not dissolve in water or methanol under the operating conditions. Four different membrane types (110, 135, 165, and 195 °C) were boiled in methanol solution (0.75 mol/L; 70 °C). All mass losses were below 1% wt. (including the standard deviation). It can be assumed that the mass loss is caused by the initial humidity in the membrane at room temperature. Based on these results, it can be expected that the membranes are stable in methanol solution at 70 °C. Furthermore, according to the results of Moore and Martin [24], it is further expected that membranes that are produced with additional solvents with high boiling points (DMAc, DMF, DMSO) will also not dissolve in methanol solution as well. Thus, these membrane types were not tested for dissolution. Following the dissolution tests, the water uptake behavior of free-standing membrane samples is important. Figure 1 shows the swelling behavior of all fabricated membrane types. The scale of the results is in accordance with those published earlier in the literature, which range from 23% to 26.5% of mass-based water uptake [29,30]. It is clear that higher temperatures in thermal treatment lead to lower water uptake. Furthermore, there is also a clear trend in terms of additional solvents. The higher the boiling point of the additional solvent (DMSO: 189 °C, DMAc: 165 °C, DMF: 153 °C), the higher the water uptake of the membranes. Further correlations to the degree of crystallinity will be discussed later.

Water uptake can be an important factor when spray-deposited membranes and CCMs are tested for long-term durability, as the swelling and de-swelling of membranes can influence the membrane–electrode interface and cause delamination between both if membranes and electrodes swell differently. Furthermore, it is to be expected that membranes with higher water uptake could also allow higher methanol permeation rates, as water and methanol are very similar to each other. All membranes except M(110) have lower degrees of water uptake than Nafion 115. Whether the degrees of water uptake are favorable in terms of protonic conductivities or overall cell performance will also be discussed later.

When considering CCMs, it is crucial to have an understanding of the topology of the membranes, as this has an impact on the membrane–electrode interface and potentially the mechanical properties. Different materials, such as glass-fiber-reinforced PTFE foils, pure PTFE foils, glass plates, aluminum foils, polyimide foils (Kapton), and polyethylene foils, were tested as substrates. Several substrates enabled the fabrication of visually smooth membrane layers. With all except the glass-fiber-reinforced PTFE foils, the adhesion of the fabricated membrane layers was too strong to detach the finished membrane without ripping it. Thus, the glass-fiber-reinforced PTFE foil was chosen as the standard for all membrane fabrication processes.

**Figure 2:**
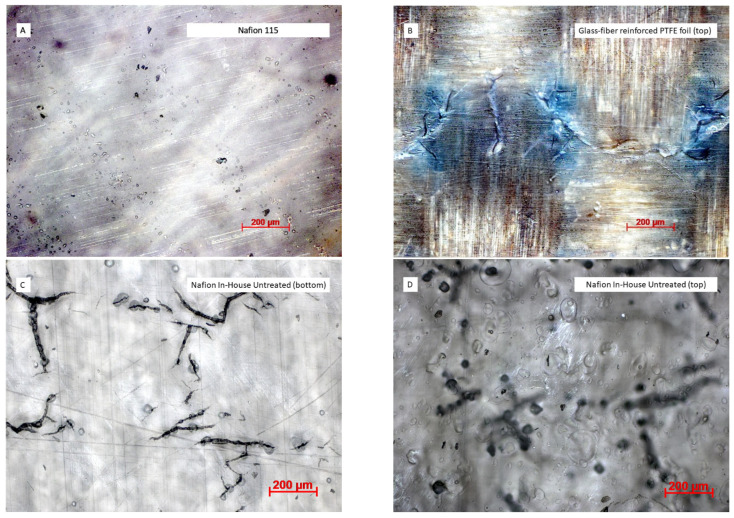
Microscopic images of Nafion 115 (**A**), glass-fiber-reinforced PTFE foil used as a substrate (**B**) and in-house fabricated membrane M(110) (**C**,**D**).

Figure 2 shows a microscopic comparison of commercial Nafion 115 (A), in-house fabricated Nafion membrane M(110) (C and D), with equivalent thickness to Nafion 115, and glass-fiber-reinforced PTFE foil, which was used as a substrate for the spray-deposition process (B). Nafion 115 shows streaks on both sides, which are presumably a result of the extrusion cast process [31]. In addition, the membrane contains air entrapments and impurities that could not be classified.

In comparison, spray-deposited membranes show no streaks, as the Nafion solution is nebulized into droplets at a µm-scale and deposited with an omnidirectional spray-pattern. However, the spray-deposited membrane samples show several cracks in a patterned order. This applies to all membrane samples—untreated, thermally treated, or with additional solvents. An analysis of the substrate revealed that these cracks are caused by the woven tissue structure of the glass-fibers within the foil. Despite crack formations at the bottom of the membranes, an analysis of the top (Figure 2D) reveals that these cracks only occur one-sidedly.

There does not seem to be through-plane crack propagation. This will not be a problem when the entire CCM is produced by spray deposition, as the membrane will be deposited onto the first electrode. Still, with the current set-up of self-standing membranes, there are cracks that must be considered prior to fuel cell operation. Cracks can have a negative impact on mechanical stability, hydrogen or methanol permeation, or cause short-circuits.

**Figure 3:**
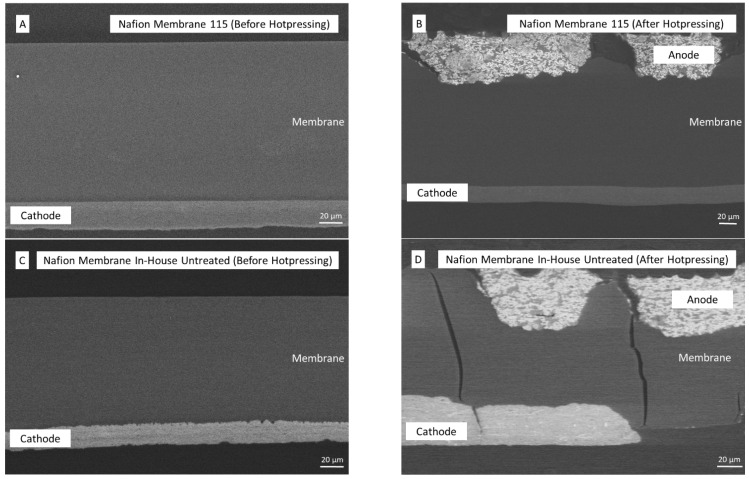
SEM images of commercial Nafion 115 (**A**) and in-house-fabricated membrane coated with electrodes (**C**) and Nafion 115 (**B**) and in-house-fabricated membrane sample after coating and hot-pressing with anode GDEs (**D**).

Cross-sectional SEM analyses can give further insight into membrane thickness distribution and the role of cracks with regard to short circuits. Figure 3 shows SEM images of different stages of the MEA fabrication with Nafion 115 and spray-deposited M (110). Cracks at spray-deposited membrane surfaces cannot be detected in the cross-section (Figure 3C).

Furthermore, when comparing commercial to spray-deposited membranes at the membrane–electrode interfaces, the commercial membrane surface seems to be smooth, whereas the spray-deposited membrane shows some surface roughness (Figure 3A,C). This is in accordance with microscopic examination of the membrane surfaces (Figure 2). Nebulized Nafion solution drops, generated by ultra-sonication, dry instantly when deposited onto the previous layer.

This might be a reason for the surface roughness, as there is no opportunity for polymer-chain movement under the chosen conditions (110 °C). On the other hand, when not produced as a self-standing membrane but rather spray deposited onto an electrode, spray depositing single droplets of Nafion solution may be beneficial, as they will allow ideal coverage of the entire electrode surface, even if the catalyst carrier shows some surface roughness. This could potentially make decal-transfer processes obsolete.

Figure 3 shows further microscopic images of the cross-section after hot-pressing of the anode GDEs onto the opposite side of the membrane (Figure 3B,D). Here, another disadvantage of the hot-pressing or decal transfer process is exposed.

**Figure 4:**
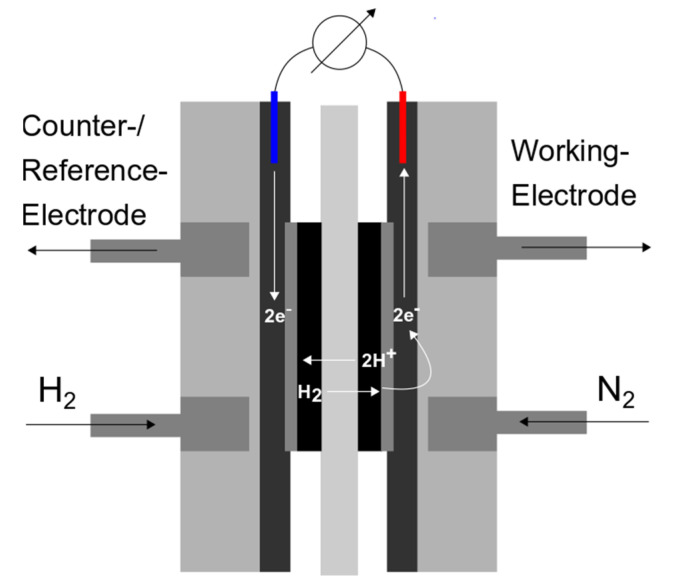
Schematic drawing of in situ linear sweep voltammetry for hydrogen permeation measurements (Anode: red, Cathode: blue).

While the spray-coated side (cathode) of the membrane is still intact, both membranes experience deformations on the other side (anode) due to the hot-pressing of a GDE. Furthermore, the spray-deposited membranes show some cracks. Additionally, due to deformation, some parts of the anodes are not properly attached to the membrane. This problem can be avoided when the entire CCM is spray deposited. These through-plane cracks could potentially entail problems regarding fuel crossover (e.g., hydrogen or methanol). A method to determine hydrogen permeability and detect membrane defects (e.g., short circuits or holes) is in situ linear sweep voltammetry (Figure 4) [18,32].

**Figure 5:**
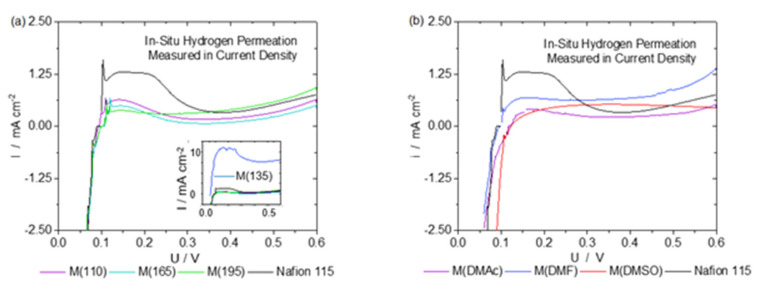
Linear sweep voltammograms (sweep rate 5 mV s^−1^) of membranes with different thermal treatments (**a**) and additional solvents (**b**). All membranes except for membrane 135 °C are in the same range of hydrogen permeability measured as current density.

While purging the anode with hydrogen and the cathode with nitrogen, a voltammetric linear sweep was applied to the cathode (0–0.6 V). Hydrogen that permeates from the anode through the membrane is oxidized at the cathode. This electrochemical conversion can be measured as a current and represents a scale for hydrogen crossover through the membranes [32]. Figure 5 shows the results of in situ hydrogen permeation measurements of all membranes.

All membranes, except for M(135) (Figure 5 inset, blue line), show a similar permeability towards hydrogen as commercial Nafion 115. Several repetitions of the same experiment with other M(135) samples confirmed the first result. All others are more or less within the same region of less than 1.25 mA cm^−2^. This is in accordance with the results published by Klingele et al., wherein the data show an increasing trend, but the permeation current density does not exceed 1.5 mA/cm^2^. Both results are in concordance, even though the membranes fabricated by Klingele et al. were sprayed onto a GDE (electrode-supported MEA) [18]. This is also comparable to results presented by Bayer et al., where the crossover increased rapidly and then leveled off around 2.5 mA/cm^2^, just as in our case [19]. A limitation of this method is that the current measured changes slightly with potential, while showing very low values for all potentials. We are presenting these results for transparency instead of hiding them by giving permeation currents only for one potential. We would like to stress that all membrane samples only allow such low permeabilities, even though they showed some cracks in the SEM analysis. This leads to the conclusion that the extent of crack size and density could play a minor role in terms of hydrogen crossover, or that only a tiny fraction of the membranes have cracks that were then coincidentally detected in the samples. Regarding thermal treatment or the addition of high-boiling point solvents, hydrogen permeability measurements show no benefit in thermal treatment or in the addition of extra solvents during the production process. Another important factor that can help determine the effects of the membrane production method, thermal treatment, or modification by high-boiling point solvents are ohmic losses.

These were determined by measuring high-frequency resistances via ac impedance spectroscopy in a half-cell setup (anode: methanol; cathode: hydrogen). The resulting data were refined by means of a two-pole Hilbert transformation algorithm (ZHIT) [33]. The ohmic losses were determined at the interception of the Nyquist-plots with the x-axis. To eliminate cell hardware contributions, the ohmic resistances of the GDLs and flow-fields were measured separately. The sum of these was 0.060 ± 0.001 Ω cm^2^, which was then subtracted from the high-frequency resistance. The results shown in Table 1 represent the ohmic resistances of the entire CCM, including the anode, membrane, cathode and contact resistances between the flow-fields and GDLs, GDLs and electrodes and the electrodes and the membrane.

With the current setup of consecutively spraying membranes and electrodes, it is not possible to distinguish between membrane and electrode contributions. To discuss the membrane separately, the following calculations are employed as a first approximation. Slade et al. experimentally measured the protonic conductivities of Nafion 115 at 70 °C in a fully hydrated state and determined it to be 0.115 ± 0.009 S cm^−1^ [34]. At a swollen thickness of 140 ± 9 µm, this corresponds to an ohmic resistance of 0.12 ± 0.01 Ω cm^2^. The subtraction in (1) then leads to the sum of ohmic resistances of the electrodes and contact resistances, as well as other sources of ohmic losses not considered here, and is 0.08 ± 0.02 Ω cm^2^. This value is used for (2) to calculate the membrane contributions only. The results are shown in Table 2, below.

(1) $$R_{CCM}-R_{Nafion115}=R_{Remaining}$$

(2) $$R_{CCM}-R_{Remaining}=R_{Membrane}$$

Membranes M(110)-M(195) show impedances within the same range (including standard deviation) or below Nafion 115. The same applies for membrane M(DMSO) but not M(DMAc) or M(DMF). To discuss the thermally treated membranes first, the overall range of ohmic resistances is roughly comparable to data shown in the literature [17,19,20,35]. However, an attempt to compare thickness-independent conductivities seems difficult, as some authors only show high-frequency resistance (HFR), which does not exclude contributions from electrodes, cell hardware, and contact resistances [35]. Others only measure the membrane resistances, but under conditions that are not comparable to those chosen in this work, but rather with humidified gases that result in higher ohmic resistances and lower protonic conductivities [17,19]. Considering membranes produced with additional high-boiling point solvents, there is a clear trend of increasing impedances from DMSO to DMF, which is inverse to the water uptake values shown in Figure 1b. The lower the water uptake, the higher the ohmic resistances. This result is predictable. Overall, it can be concluded that EIS measurements provide no data that would hint towards improved membrane characteristics in the case of thermal treatment or additional solvents.

**Figure 6:**
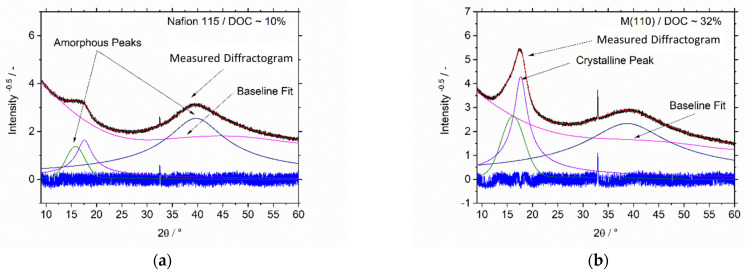
Fitted wide angle X-ray diffractograms (XRD) of (**a**) Nafion 115 and (**b**) in-house-fabricated membrane M(110) with fits showing the peaks for the crystalline and amorphous regions of the Nafion polymer chains [36].

In contrast, adding solvents with high boiling points, which is supposed to enhance polymer-chain mobility and thus crystallinity, has a somewhat negative effect on the ohmic resistances of the membrane samples. The reason for this could be a chemical reaction of the solvents with Nafion that will be discussed later. To clarify whether the crystallinity of the membranes is the reason for differences in water uptake and ohmic resistance, XRD of all samples were measured. Figure 6 shows the diffractograms of commercial Nafion 115 and spray-deposited membrane M(110). The figures show the difference between measured and calculated intensities (blue), observed measurements (black) and calculated profile (red); a baseline fit was generated by a fourth order Chebyshev polynomial (pink), while the peaks of amorphous and crystalline regions were assigned on the basis of the findings of Gebel et al. [22] and fitted by a pseudo-Voigt function (full width at half maximum (*FWHM*)) with a refinement factor (0–1) to define the fit as either a Gaussian or Lorentzian type. The determined peaks and peak areas could then be used to calculate the degree of crystallinity (*DOC*) of the membrane samples, using the following equation:

(3) $$DOC=\frac{{PA}_{Crystalline}}{{PA}_{Crystalline}+{PA}_{Amorphous}}\times 100\%$$

where *PA* is the peak areas of the different phases in the polymer bulk.

It must be pointed out that the resulting values are only a relative degree of crystallinity, as they represent a ratio between crystalline and amorphous regions determined by XRD. Nevertheless, the resulting data are a first step towards an understanding of the relationship between crystallinity and the specific characteristics of fabricated membranes. The results of the degree of crystallinity calculations are shown in Table 3 and Table 4.

Nafion 115 has a significantly lower degree of crystallinity than commercial PTFE, which has been used as a reference to identify the crystalline regions of the polymer backbone. This result is to be expected, as PTFE does not contain a sulfonic acid-based side disturbing the formation of crystalline regions. Moreover, the value of 10% corresponds to other results in the literature, where values of around 11–12% were found [21].

However, membranes produced by spray deposition (untreated, thermally treated, or based on additional solvents) all show significantly sharper peaks in the crystalline region and also significantly higher values for their degree of crystallinity compared to commercial Nafion 115. Moreover, starting from M(135), there is a trend towards decreasing DOC with increasing annealing temperatures. This is an inverse trend to the water uptake values.

**Figure 7:**
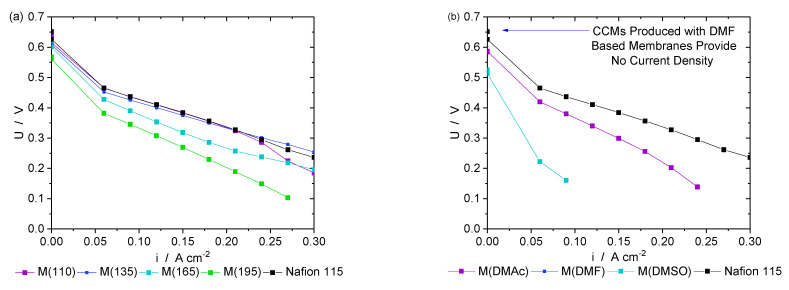
Performance tests (**a**) with different thermal treatments and (**b**) additional solvents. Standard deviations too small for proper visualization. M(110) and M(135) could match the performance reached with a commercial Nafion 115 membrane.

Additionally, all membrane types were also employed for single cell tests during DMFC operation. Figure 7 shows the polarization curves of CCMs with all in-house-fabricated membrane types in comparison to commercial Nafion 115. As expected, based on ohmic resistances, degrees of crystallinity, and water swelling values, MEAs produced with membranes based on additional solvents performed much worse than an MEA with Nafion 115. DMF-based membrane samples could not be tested at all, as the voltage dropped below the minimum limit of 100 mV at the first point (60 mA cm^−2^).

Only membranes M(110) and M(135) show polarization curves that match the performance measured with a Nafion 115 membrane throughout most of the tested current density range. This is not the case for CCMs based on membranes M(165) and M(195). The polarization curves are in accordance with the water uptake and degrees of crystallinity. The higher the water uptake, the better the performance in a single cell test. Nafion 115, M(110), and M(135) all show the same water uptake values (within standard deviation) as well as comparable polarization curves. The polarization curves only become worse with membranes treated at higher temperatures that also take up less water. A clear relationship between water uptake/performance and degree of crystallinity could not be established.

This behavior of the membranes is not reflected by the results of the EIS measurements because the tests were conducted in a single cell setting where all components contribute to the ohmic resistances. The subtraction of their contributions, including the standard deviations, leads to uncertainties that do not allow for a distinctive comparison and determination of thermal impact on the membrane resistances.

Nevertheless, the relationship between thermal treatment and lower performances of CCMs supports the findings of Jung and Kim, who thermally treated Nafion 117 and tested it for DMFC operations [37]. Jung and Kim explain their findings with XRD measurements whereby they could detect an increasing peak in the crystalline region. However, Jung and Kim only analyzed their data qualitatively, and not quantitatively. Therefore, for their case, it is not clear if the annealing temperature actually increases the degree of crystallinity. This explanation could not be confirmed with our own results, as the discussed membrane types all show higher degrees of crystallinity than Nafion 115 but behave differently in single cell tests.

Decreasing the degree of crystallinity with increasing annealing temperature corresponds to the results from Luan et al. [36]. The authors show that annealing temperatures above 150 °C start decreasing the degree of crystallinity. Based on the results from Page et al. [38], they argue that small crystallites with an imperfect structure start to melt, which enables the sulfonic acid groups of the side chains to move. This movement is, however, supposed to disable the formation of new crystallites based on the polymer main chain, which causes a decrease in crystallinity.

However, the free movement of sulfonic acid groups should also lead to the growth of existing or the formation of new clusters that should yield improved protonic conductivities. Luan et al. used small angle X-ray scattering (SAXS) and EIS measurements to confirm this hypothesis, as they were able to detect larger and more organized clusters, as well as increasing protonic conductivity with increasing annealing temperature [36]. A comparison of solution cast membranes and extruded membranes was done by Collette et al. [39]. As outlined above and shown in Table 2, the ohmic resistances determined in this work are in the range of 0.10 Ω cm^2^ ± 0.02 Ω cm^2^ have standard deviations of 0.02 Ω cm^2^ so they can be considered as equal within the margin of error. However, if the protonic resistance is increased, as in the case of M(DMAc), M(DMSO), and M(DMF), the performance significantly decreases. Conductivities significantly higher than for commercial Nafion 115 were not obtained. We would expect improved single cell performances in this case. The performance differences among membranes with similar conductivities shown in Figure 7 are expected to be due to experimental details other than conductivity.

Luan et al. left annealed membranes in the oven to cool down in a controlled manner for 3–4 h [36]. In our study, membranes were removed from the oven after the annealing time of one hour was over and thus were instantly cooled down to room temperature. Thus, we could expect that the polymer sidechains of the melted crystallites were first moving freely due to the high temperature and were then “frozen” without any cluster formation when immediately cooled down to room temperature.

For membranes based on high-boiling point solvents, we would consider the following explanations:

Hongsirikarn et al. showed that ammonia poisoning of Nafion membranes can drastically decrease proton conductivity [40]. This is assigned to a transfer from the protonated form to a salt form with ammonium (NH_4_^+^) as a cation. Applying these findings to the given results in this work, it could be expected that membrane poisoning can occur. Detailed explanations of possible side reactions are listed in Table 5.

**Figure 8:**
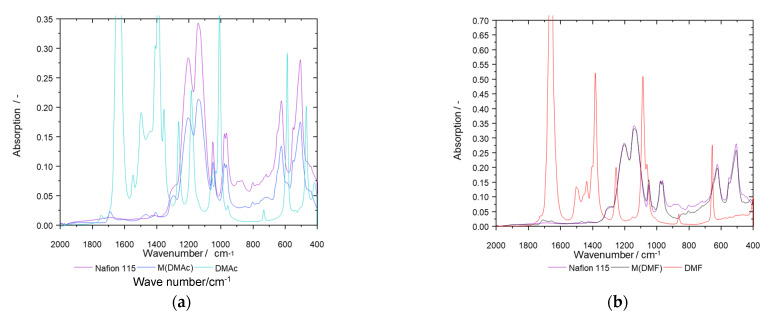
(**a**) IR spectra of Nafion 115 and membrane samples prepared with additional DMAc and DMAc and (**b)** IR spectra comparison of Nafion 115, M(DMF), and DMF.

The idea of membrane poisoning could be partly confirmed for membranes based on DMF and DMAc. Figure 8 shows an overlap of the IR spectra of the solvent-based membrane, Nafion 115 and each solvent used for it. M(DMF) shows absorption peaks at 1711 and 1654 cm^−1^. These peaks cannot be found in Nafion 115 but in DMF. The same applies for M(DMAc) where peaks at 1689, 1469, and 1407 cm^−1^ were detected that do not occur in Nafion 115 but in DMAc. Furthermore, when compared to membranes produced by spray deposition but without additional solvents, these peaks could also not be detected in the IR spectra. This clearly shows that some solvent remains in the membrane. With M(DMF), the X-ray diffractogram shows an additional peak at 2Θ = 22° that does not belong to Nafion. All these results indicate that traces of the solvents could be poisonous for the membrane, as they apparently do not leach out. These explanations do not work for M(DMSO), as there are no signs of membrane poisoning by DMSO.

## 4. Conclusions

In this work, we were able to show that it is possible to produce membranes and CCMs based on ultrasonic spray deposition alone. Membranes have equivalent properties in terms of thickness, ohmic resistances, and performance compared to Nafion 115. Membranes produced with ultrasonic spray deposition allow hydrogen permeabilities below 1.25 mA/cm^2^. They also have a degree of crystallinity above 30%, which decreases with increasing annealing temperature. Contrary to what is shown in the literature, higher annealing temperatures did not lead to improved CCM performances due to higher conductivities. When adding additional solvents such as DMF or DMAc for spray deposition, the chosen conditions can lead to the decomposition of solvents, which causes membrane poisoning. This work demonstrates that hot-pressing and transfer processes are obsolete for CCM manufacturing. In addition, these findings will allow further modifications, such as membrane reinforcements or interlayers as permeation blockers.

## Figures and Tables

**Table 1:** Ohmic resistances of the CCMs (including contact resistances) based on commercial Nafion 115 and membrane samples produced with spray-deposition, different thermal treatments, and additional solvents.

Membrane Type	Ohmic Resistance *R* ± Standard Deviation *σ*/Ω cm^2^
Nafion 115	0.21 ± 0.01
M(110)	0.18 ± 0.02
M(135)	0.16 ± 0.02
M(165)	0.18 ± 0.02
M(195)	0.18 ± 0.01
M(DMSO)	0.23 ± 0.04
M(DMAc)	0.30 ± 0.02
M(DMF)	2.18 ± 0.08

**Table 2:** Approximated ohmic resistances of Nafion 115 and spray-deposited membranes.

Membrane Type	Ohmic Resistance *R* ± Standard Deviation *σ*/Ω cm^2^
Nafion 115	0.12 ± 0.01
M(110)	0.10 ± 0.02
M(135)	0.08 ± 0.02
M(165)	0.10 ± 0.02
M(195)	0.10 ± 0.01
M(DMSO)	0.15 ± 0.04
M(DMAc)	0.22 ± 0.02
M(DMF)	2.10 ± 0.08

**Table 3:** Degree of crystallinity of a commercial Nafion 115 membrane and membrane samples produced with spray deposition and different thermal treatments.

Membrane Type	Degree of Crystallinity/%
Nafion 115	10
Commercial PTFE	33
M(110)	33
M(135)	34
M(165)	31
M(195)	23

**Table 4:** Degree of crystallinity of a commercial Nafion 115 membrane and membrane samples produced with spray deposition and additional solvents.

Membrane Type	Degree of Crystallinity/%
Nafion 115	10
Commercial PTFE	33
M(DMSO)	36
M(DMAc)	33
M(DMF)	44

**Table 5:** Solvents and their reactions under given conditions.

Solvent	Reaction
Dimethylacetamide (DMAc) [41]	It is reported that DMAc, under strong acidic conditions, undergoes hydrolysis, releasing acetic acid and dimethylamine. Alternatively, it can undergo alcoholysis under acidic conditions, whereby an acetic ester and dimethylamine are released. A dimethylamine-based Nafion salt would have much lower conductivities.
Dimethylformamide (DMF) [42]	DMF can decompose into carbon monoxide and dimethylamine at its boiling point (153 °C). This temperature was not reached during membrane manufacturing. Nevertheless, decomposition can be catalyzed under acidic conditions.
Dimethyl sulfoxide (DMSO) [43]	DMSO can undergo a disproportionation reaction to dimethyl sulfide and dimethyl sulfone at 90 °C. To us, it is not clear what could potentially happen after this step to cause membranes to have such a high ohmic resistance and the resulting CCM to perform poorly.

## Data Availability

Data are contained within the article.
